# C-reactive protein velocity discriminates between acute viral and bacterial infections in patients who present with relatively low CRP concentrations

**DOI:** 10.1186/s12879-021-06878-y

**Published:** 2021-12-04

**Authors:** Daniel Bernstein, Dan Coster, Shlomo Berliner, Itzhak Shapira, David Zeltser, Ori Rogowski, Amos Adler, Ora Halutz, Tal Levinson, Omri Ritter, Shani Shenhar-Tsarfaty, Asaf Wasserman

**Affiliations:** 1 Joyce & Irving Goldman Medical School, Faculty of Health Sciences, Ben-Gurion University of the Negev, Beersheba, Israel; 2 Blavatnik School of Computer Science, Tel-Aviv University, Tel-Aviv, Israel; 3 Departments of Internal Medicine “C”, “D” and “E”, Tel-Aviv Sourasky Medical Center, and Sackler Faculty of Medicine, Tel-Aviv University, 6 Weizmann Street, 64239 Tel Aviv, Israel; 4 Clinical Microbiology Laboratory, Tel-Aviv Sourasky Medical Center, Tel-Aviv, Israel; 5 Infectious Diseases Unit, Tel-Aviv Sourasky Medical Center, Tel-Aviv, Israel; 6 Department of Emergency Medicine, Tel-Aviv Sourasky Medical Center, Tel-Aviv, Israel

**Keywords:** Bacterial, Viral, C-reactive protein, Velocity, Differential diagnosis, Infection

## Abstract

**Background:**

To assess the utility of C-reactive protein (CRP) velocity to discriminate between patients with acute viral and bacterial infections who presented with relatively low CRP concentrations and were suspected of having a bacterial infection.

**Methods:**

We analyzed a retrospective cohort of patients with acute infections who presented to the emergency department (ED) with a relatively low first CRP measurement (CRP1) ≤ 31.9 mg/L and received antibiotics shortly after. We then calculated C-reactive protein velocity (CRPv), milligram per liter per hour, for each patient based on CRP1 and the second CRP value (CRP2) measured within the first 24 h since admission. Finally, we compared CRPv between patients with bacterial and viral infections.

**Results:**

We have presently analyzed 74 patients with acute bacterial infections and 62 patients with acute viral infections at the mean age of 80 and 66 years respectively, 68 male and 68 female. CRP1 did not differ between both groups of patients (16.2 ± 8.6 and 14.8 ± 8.5 for patients with viral and bacterial infections respectively, p value = 0.336). However, the CRP2 was significantly different between the groups (30.2 ± 21.9 and 75.6 ± 51.3 for patients with viral and bacterial infections respectively, p-value < 0.001) and especially the CRPv was much higher in patients with acute bacterial infections compared to patients with acute viral infections (0.9 ± 1.2 and 4.4 ± 2.7 respectively, p-value < 0.001).

**Conclusion:**

CRPv and CRP2 are useful biomarkers that can discriminate significantly between patients who present with acute bacterial and viral infections, and relatively low CRP concentration upon admission who were suspected of having a bacterial infection.

**Supplementary Information:**

The online version contains supplementary material available at 10.1186/s12879-021-06878-y.

## Background

C-reactive protein (CRP) is an established biomarker for the assessment of inflammation and its severity in patients who present to medical facilities [1, 2] and can be used as a reliable, fast and inexpensive indicator of the infection type [3].

Several studies showed that higher CRP values strengthen the differential diagnosis of acute bacterial infections over acute viral ones. However, the exact cut-off is still disputable and many different CRP values were suggested to indicate a bacterial infection (such as 10, 20, 40, 60 and 100 mg/L) [1, 4–7]. This highlights the difficulty for physicians in using CRP for the diagnosis of patients with bacterial infections presenting with relatively low CRP concentrations. Previous studies showed that patients who presented with relatively low CRP values constitute a substantial fraction of the admissions to the emergency department (ED) with acute infectious disease and have a high potential to deteriorate quickly. Wassermann et al. showed that 19.4% of patients admitted to ED with relatively low CRP levels (< 31.9 mg/L) and their discharge diagnosis was sepsis, died within 1 week of hospitalization [8]. Levinson et al. presented that 6% of patients diagnosed with gram-negative bacteremia and had an initial low CRP measurement (< 30 mg/L) died within 1 week of hospitalization [9]. Feigin et al. suggested that presentation to the internal medicine department with a very low concentration of CRP (< 0.05) does not exclude the existence of significant acute morbidities [10]. These studies emphasize the importance of early diagnosis in acutely ill patients presenting with relatively low CRP values.

Studies have shown the usefulness of sequential measurements of CRP as a tool in the follow-up of different conditions such as community-acquired Pneumonia, ventilator-associated pneumonia, bloodstream infection and sepsis [9, 11]. The ratio between consecutive CRP measurements was suggested to assess the patient prognosis or the appropriateness of antibiotic therapy. Other studies tried to use the kinetics of CRP for the microbial etiology diagnosis, specifically in the early discrimination between acute viral and bacterial infections. Our own study by Paran et al. presented the possibility to use CRP velocity as a diagnostic tool in patients with bacterial febrile diseases [12]. More recently, Coster et al. presented the usefulness of CRP trend, based on the rate of change between the first and second CRP measurements taken after admission, demonstrating the potential utility of this biomarker for the differentiation of viral versus bacterial infections [13]. These findings strengthen our hypothesis that utilizing CRP kinetics at the first hours upon admission to the medical center can improve the distinction between acute viral and bacterial infections, as the rate of CRP concentration change holds more information regarding the inflammatory process over time than a single CRP measurement. Given good discrimination by CRP kinetics among patients presenting with relatively low CRP values, which are not indicative of infection type, could help in avoiding misuse of antibiotics.

## Methods

### Study design and setting

We conducted a historical cohort study of patients admitted through the ED in Tel Aviv Sourasky Medical Center (TASMC), Israel, between April 2011 and March 2019. We proposed investigating our assumption by looking at patients with viral and bacterial infections who presented with relatively low first CRP measurement (CRP1) and received antibiotics soon after. This cohort enabled us to evaluate the differentiation ability of CRPv when the CRP1 upon admission was not indicative of infection type, and yet, physicians decided on treatment with antibiotics. This clinical scenario reflects a setting where a differentiation biomarker could be highly significant and prevent inappropriate antibiotic treatment. Hospital records were reviewed manually in order to apply the exclusion criteria.

### Patients

All the patients had a relatively low CRP1 ≤ 31.9 mg/L, upon admission and a positive lab test for either a viral or bacterial infection. This relatively low CRP concentration cut-off represents the mean CRP of apparently healthy individuals + 3 standard deviation. It was suggested by Wasserman et al., based on a relatively large cohort of individuals who were screened as part of a routine annual check-up [8].

#### Inclusion criteria

We included all patients admitted to the general ED in TASMC, Israel, who are adults aged ≥ 18. We could not include younger patients in this study because they are admitted to a separate pediatric ED in TASMC, and we did not have access with the current Helsinki approval to their medical records. Bacterial infections were identified by a positive blood culture for a single bacteria species that is likely to cause infection and not be a contaminant (defined as blood culture results of *Diphtheroids *spp, *Coagulase-negative Staphylococcus*, *Streptococcus viridans group* and a result of *Gram-positive bacilli*). Viral infections were identified by either a positive PCR for a virus or an immunoglobulin test indicative of a single viral species.

To link between the CRP measurement and the infectious diagnosis we only included patients whose positive blood culture was taken within 1 day or positive viral test within 5 days of the first CRP measurement. The time difference between CRP1 to the second CRP measurement (CRP2) was less than 24 h.

#### Exclusion criteria

Patients who were not treated with antibiotics between CRP1 and CRP2 or received antibiotics during the 24 h before admission.

Patients with co-infections, defined as having both a bacterial and viral positive test.

**Fig. 1:**
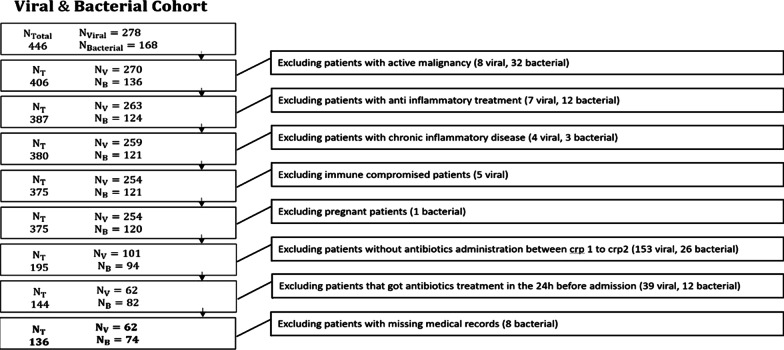
Study design

Patients with active malignancy or active inflammatory disease, for example, Systemic lupus erythematosus, Inflammatory bowel disease etc. Patients who were treated with anti-inflammatory medications or immunocompromised patients. Pregnant women and patients with missing medical records were excluded as well (Fig. 1).

### Laboratory methods

Wide range (WR) CRP measurements were done by ADVIA 2400 (Siemens Healthcare Diagnostics Inc., Tarrytown, NY, USA). The ADVIA^®^ Chemistry WR-CRP method measures CRP in the serum and plasma by a latex-enhanced immunoturbidimetric assay.

### Computational methods

CRP velocity (CRPv) was defined as the difference between the second CRP measurement (CRP2) and the first measurement (CRP1), divided by the time difference between the tests (Δt CRP1 to CRP2, hours).

$$\mathrm{C}RPv=\frac{CRP2-CRP1}{\Delta\mathrm{t \,\,CRP}1\,\,\mathrm{to\,\, CRP}2}$$

Estimated CRP (eCRP) was defined as expected CRP in apparently healthy individuals based on age and sex. eCRP was calculated based on the data of Tel Aviv Medical Center Inflammation Survey. First, we divided the cohort by sex and then we calculated the mean CRP by age groups of 5 years (starting with individuals 20–25 years old to 75 years). There was a relatively small number of subjects in the male and female group above the age of 75, hence, we calculated their mean CRP concentration and considered it as the eCRP of all the patients above the age of 75 years. In addition, the healthy cohort did not include subjects younger than 20 years, so we clinically estimated the eCRP value of this age group (Our cohort had only one female patient younger than 20 and her eCRP value was 1.5 mg/L). The eCRP values of each age group are reported on Additional file 1: Table S1. The purpose of calculating eCRP is to estimate the CRP level of the patient in the healthy condition in the initiation of his pathological course. Estimated CRP velocity (eCRPv) was defined as the difference between CRP1 and eCRP divided by the time difference between the beginning of symptoms and CRP1 test (Δt onset of symptoms to CRP1, hours) was defined as eCRPv.

The exact timing from the onset of symptoms was estimated based on the medical record of each patient’s admission file.

$$\mathrm{eC}RPv=\frac{CRP1-\mathrm{eCRP} }{\Delta \mathrm{t \,\,between \,CRP}1\,\mathrm{and\, symptoms \,onset}}$$

The recorded antibiotic time was used to calculate the Δt Antibiotic; the time difference between the CRP1 measurement and antibiotic administration.

$$\Delta\mathrm{t \,Antibiotic}={{t}_{Antibiotic \, Administration}}-{{t}_{CRP1}}$$

For cases in which the administration time of antibiotic in ED was missing, we considered the time of admission to ward to be the antibiotic administration time.

### Statistical analysis

Categorical variables were reported as numbers and percentages. The continuous variables were reported as means with standard deviations. To compare the distributions of different features on the bacterial and the viral groups we used the non-parametric test of Mann–Whitney (MW). In order to compare the sex categorical feature, we used the Pearson’s Chi-squared test (Chi^2^). To test the performance of the numerical parameters (e.g., eCRP, CRP1, CRP2, $\mathrm{eC}RPv$, $\mathrm{C}RPv$, etc.) as biomarkers for classification, we used receiver operator characteristic curve (ROC) analysis and calculated the area under the curve (AUC) and for each parameter. Standard non-parametric bootstrapping of 1000 samples was used to generate the 95% confidence intervals. The statistical analysis was performed using the Python programming language version 3.5.2 and the packages SciPy, NumPy and scikit-learn.

## Results

Applying our inclusion criteria our study covered 446 adult patients, 168 with bacterial infections and 278 with viral infections. Applying our exclusion criteria, the final cohort for analysis covered 136 patients, 74 (54%) with bacterial infection and 62 (46%) with viral infection. Their characteristics are presented in Table 1. Specific bacterial and viral types are reported in Additional file 1: Tables S2 and S3 respectively.

**Table 1:** Demographic characteristics of the bacterial and viral groups

Table of characteristics						
Name	Viral N = 62	Bacterial N = 74	AUC	CI	MW p value	ChiSquare p value
Age (years)	66.6 ± 18.4	80.2 ± 13.7	0.74	–	< 0.001	–
Women (%)	58.1%	43.2%	0.57	–	–	0.12
Δt Antibiotic (h)	5.4 ± 5	4.2 ± 4.2	0.58	–	0.097	–
eCRP	2.9 ± 0.3	2.8 ± 0.2	0.58	–	0.116	–
CRP1 (mg/L)	16.2 ± 8.6	14.8 ± 8.5	0.55	0.46–0.62	0.336	–
Δt onset of symptoms to CRP1 (h)	72.7 ± 104	29.1 ± 60.8	0.75	0.67–0.82	< 0.001	–
eCRPv (mg/L/h)	0.8 ± 1.6	1.2 ± 1.1	0.7	0.62–0.77	0.001	–
CRP2 (mg/L)	30.2 ± 21.9	75.6 ± 51.3	0.77	0.70–0.84	< 0.001	–
Δt CRP1 to CRP2 (h)	14.9 ± 5.9	13.1 ± 6.4	0.58	–	0.108	–
CRPv (mg/L/h)	0.9 ± 1.2	4.4 ± 2.7	0.86	0.79–0.91	< 0.001	–

Values are mean ± SD, % for women, MW p-value of the Mann–Whitney test, Chi2p value for the Pearson’s Chi-squared test. In the AUC computation, the positive class was bacterial infection for these parameters—eCRPv, CRP2, CRPv and viral infection for these parameters—CRP1, Δt onset of symptoms to CRP1, Δt CRP1 to CRP2. CRP1 and CRP2: the first and second CRP consecutive measurements after admission. Δt Antibiotic: the time difference between CRP1 and antibiotic administration time. eCRP: estimated CRP is the average CRP concentration in the healthy population compatible by age and sex. Δt Symptoms onset to CRP1: the time difference between onset of symptoms and CRP1. eCRPv: the difference eCRP and CRP1 divided by Δt onset of symptoms to CRP1. CRPv: the difference between CRP1 and CRP2 divided by the time difference between the tests

*AUC* Area under the curve, *CRP* C-reactive protein, *CRPv* C-reactive protein velocity, *eCRPv* Estimated C-reactive protein velocity

Patients with bacterial infections were older than patients with viral infections and had similar percent of females. The time difference between CRP1 measurements and antibiotic administration time (Δt Antibiotic) was similar between patients with viral and bacterial infections. The time differences between the onset of symptoms to the CRP1 measurement (Δt onset of symptoms to CRP1) was greater in patients with viral infections, compared to patients with bacterial infections (72.7 ± 104.0 and 29.1 ± 60.8 respectively, p-value < 0.001, AUC 0.75, CI 0.67–0.82). The $\mathrm{eC}RPv$ was greater in patients with bacterial infections compared to patients with viral infections (1.2 ± 1.1 and 0.8 ± 1.6 respectively, p-value < 0.001, AUC 0.7, CI 0.62–0.77). Patients with bacterial infections had higher CRP2 than patients with viral infections had (75.6 ± 51.3 and 30.2 ± 21.9 respectively, p-value < 0.001, AUC 0.77, CI 0.70–0.84). $\mathrm{C}RPv$ was significantly greater in patients with bacterial infections compared to patients with viral infections (4.4 ± 2.7 and 0.9 ± 1.2 respectively, p-value < 0.001, AUC 0.86 CI 0.79–0.91) (Table 1). $\mathrm{C}RPv$ had the highest classification quality in comparison to all the others classification parameters.

We evaluated the difference of CRPv levels between two age groups, one group above the age of 79 (median age) and the other below it. CRPv was statistically significant between the two age groups. After the stratification of the age groups to patients with viral and bacterial infections, the CRPv was not found to be statistically significant (reported in Additional file 1: Table S4). Next, we evaluated the difference of CRPv levels between genders and it was not found to be statistically significant. We also stratified genders to the former age groups (above and below the median age of 79 years) and it was not found to be statistically significant (reported in Additional file 1: Table S5). Lastly, we stratified gender to patients with viral and bacterial infections and it was found to be statistically insignificant (reported in Additional file 1: Table S6).

### CRPv performance for different Δt values in comparison to CRP1, eCRPv and CRP2

**Fig. 2:**
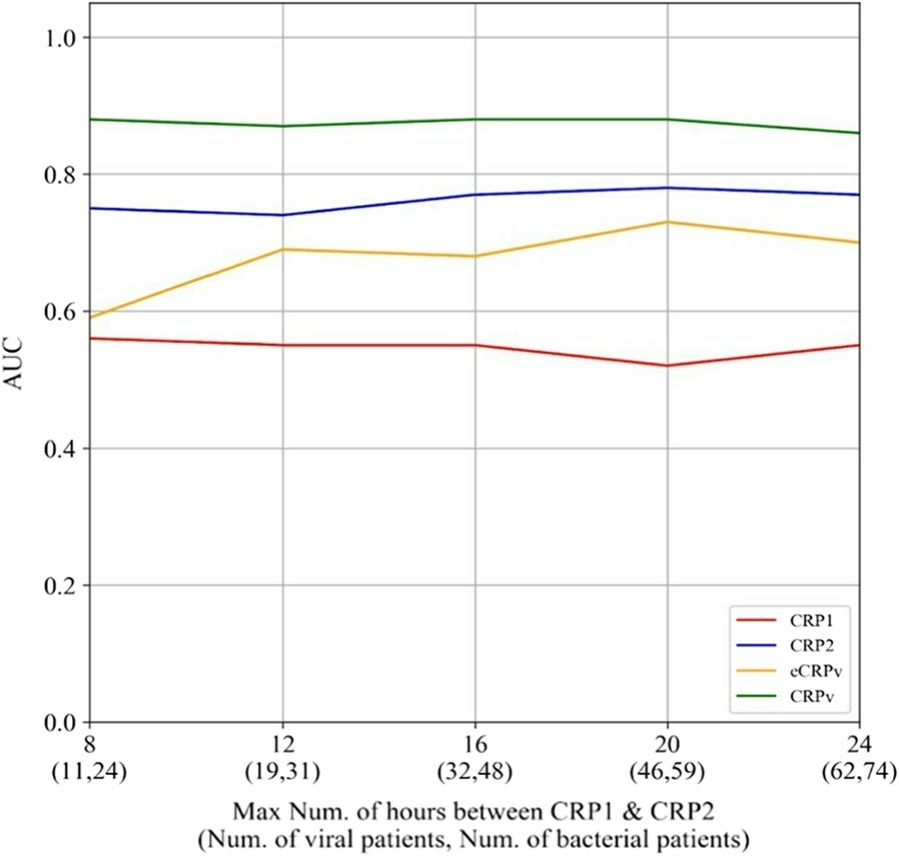
CRPv performance for different Δt values in compare to CRP1, eCRPv and CRP2. *AUC* Area under the curve, *CRP* C-reactive protein, *CRPv* C-reactive protein velocity, *eCRPv* Estimated C-reactive protein velocity

We evaluated the impact of the time difference between CRP1 and CRP2 on the classification quality in order to search for the parameter with the highest AUC on the earliest time point. We did this by reanalyzing the subgroups of patients with $\Delta\mathrm{t\, CRP}1\,\mathrm{to \, CRP}2\le X$ hours, for X values higher than 8 h (subgroups of intervals shorter than this were too small for analysis). The CRPv remained superior to all the other parameters in any time gap between the CRP1 and CRP2 measurements (Fig. 2).

## Discussion

To the best of our knowledge, this is the first study to show the usefulness of CRPv as a biomarker to distinguish between patients with acute viral and bacterial infections, who presented with relatively low CRP1 and received antibiotic treatment soon after. Our study shows that it was only CRP2, as well as CRPv that could significantly discriminate between both types of infection. Therefore, CRPv could have been used in guiding antibiotic treatment among patients with relatively low CRP1 concentrations, which and are not indicative of infection type, but make up a significant portion of ED admissions for acute infectious diseases [8]. One of strengths in our study is that we included only patients treated with antibiotics, using it as a marker for high clinical suspicion of bacterial infection. The use of antibiotic treatment in patients with proven viral disease shows that the treating physicians got no indication of infection type using the result of the first CRP test. By looking at the second CRP, taken only several hours later, they could have obtained much more information and possibly avoided antibiotic misuse.

In addition, we could show that by calculating the eCRPv upon admission, we could significantly increase the ability to differentiate between viral and bacterial infections already during the presentation to the medical facility. We do believe that this is an additional significant finding of the present study as the information of duration of symptoms at the time of CRP1 measurement is readily accessible to the treating physician and can also help in guiding antibiotic treatment.

The CRP2 and especially CRPv further helped to differ between bacterial and viral infections, with a rapid rise in CRP associated with bacterial infections. Our findings demonstrate that for each time point examined, using CRP velocity is more informative than the absolute value of the CRP measurement (e.g., the AUC of eCRPv is higher than the AUC of CRP1, and the AUC of CRPv are higher than the AUC of CRP2).

In the present study, we have calculated only the rise of CRP at the early hours following admission and not the trend as described by Coster et al. [13]. CRP trend uses all the changes in CRP concentration, including CRP decrements, which can also help in the process of differentiation between both types of infection. However, this type of analysis (CRP trend) is less practical for patients who present with relatively low CRP concentrations. It might be more relevant in individuals who present with high CRP concentrations.

Previous studies indicated that CRP levels among healthy individuals are associated with age and gender [14, 15] while a recent large study did not support that [16]. Hence, we evaluated their impact on CRPv as potential confounders. We found that CRPv was higher among adults older than 80 years old however, after stratification to infection type, there was no difference between the age groups. It was probably because patients with bacterial infections were older and their CRPv levels were found to be also higher than patients with viral infections (reported in Additional file 1: Table S4). We did not find differences in CRPv among genders, also after stratification to infection type (reported in Additional file 1: Tables S5 and S6 respectively). To the best of our knowledge, we are the first to evaluate the association of CRPv levels with age and gender.

Our study has several limitations, the main one being a retrospective study. In addition, sorting out a certain group of patients that were treated with antibiotics might impose a selection bias. However, this bias is also one of the strengths of our study since it suggests a new paradigm for avoiding antibiotics misuse. Moreover, the fact that all the patients in our cohort were treated with antibiotics could play as a confounding factor. While the patients with bacterial infections are supposed to be affected by the antibiotic treatment, the patients with viral infections are inherently unaffected. Considering Coster et al. results [13], including also patients who were not treated with antibiotics and showed that CRPv could still distinguish viral from bacterial infections, we may assume this confounding factor weakens. Next, we plan to expand our research and evaluate our findings with more diverse groups such as viral who got appropriate treatment and bacterial who did not get appropriate treatment. In addition, some possible confounders of CRP levels were not recorded in our dataset, such as race [15]. In our subsequent studies, we wish to evaluate them prospectively. To the best of our knowledge, the effect of these variables has not been researched in association with CRP kinetics.

Despite the limitations mentioned above, we believe our findings may assist in an actual clinical problem. In our cohort, the admitting physician decided to treat all patients with antibiotics, although 45% of them were later discovered to have had a viral diagnosis, showing the limits of a single relatively low CRP measurement to reduce antibiotic misuse. With the growing problem of antibiotic resistance [17–19], simple tools such as using the kinetics of CRP as shown in our study should be further explored.

## Conclusions

In patients presenting with relatively low CRP concentrations but high clinical suspicion of bacterial infection, calculating the rate of rise to first CRP and the dynamics to a second CRP measurement helps in differentiating between bacterial and viral etiologies.

## Supplementary Information

**Additional file 1.** Tables S1–S6.

## Data Availability

The data that support the findings of this study are available from the corresponding author, [T.L.], upon reasonable request.

## Abbreviations

AUC: Area under the curve
CRP: C-reactive protein
eCRP: Estimated C-reactive protein
CRPv: C-reactive protein velocity
eCRPv: Estimated C-reactive protein velocity
ED: Emergency department
WR-CRP: Wide range C-reactive protein
MW: Mann–Whitney
ROC: Receiver operator characteristic curve
