# Supplementary material for: C-reactive protein velocity discriminates between acute viral and bacterial infections in patients who present with relatively low CRP concentrations
Source: BMC Infect Dis. 2021 Dec 4;21:1210. doi: 10.1186/s12879-021-06878-y (PMC8643010; doi:10.1186/s12879-021-06878-y)
Supplement: Supplementary file 1 — Additional file 1. Tables S1–S6. [file 12879_2021_6878_MOESM1_ESM.docx]

# **Additional file 1**

**Table S1:**

| eCRP values | | | | |
| --- | --- | --- | --- | --- |
| Age Interval | Males | | Females | |
|  | n | Mean | n | Mean |
| < 20* | - | 3.5 | - | 1.5 |
| (20, 25] | 263 | 3.676612167 | 504 | 1.537853175 |
| (25, 30] | 714 | 3.714471989 | 1306 | 1.869887749 |
| (30, 35] | 1031 | 3.428701261 | 2322 | 2.147940568 |
| (35, 40] | 1328 | 2.895083584 | 3365 | 2.291810104 |
| (40, 45] | 2153 | 3.132661217 | 4725 | 2.27003545 |
| (45, 50] | 2492 | 3.256052889 | 4811 | 2.394020786 |
| (50, 55] | 2325 | 3.013667527 | 4188 | 2.472139446 |
| (55, 60] | 2026 | 3.1228692 | 3842 | 2.456652004 |
| (60, 65] | 1018 | 3.359552063 | 2457 | 2.483063899 |
| (65, 70] | 260 | 2.802730769 | 889 | 2.197577053 |
| (70, 75] | 82 | 2.965121951 | 243 | 2.773880658 |
| > 75** | 22 | 3.021954545 | 144 | 2.72654375 |

*****The healthy cohort did not include subjects younger than 20 years, so we clinically estimated the eCRP value of this age group.

******There was a relatively small number of subjects in the male and female group above the age of 75, hence we calculated their mean CRP concentration and considered it as the eCRP of all the patients above the age of 75.

**CRP- C-reactive protein**

**eCRP- Estimated C-reactive proteinTable S2:**

| Number of patients with each bacterial species | | |
| --- | --- | --- |
| Gram Negative | Aeromonas | 2 |
|  | E. coli | 33 |
|  | Enterobacter | 2 |
|  | Klebsiella oxytoca | 7 |
|  | Proteus | 4 |
|  | Pseudomonas | 4 |
|  | Shwanella algae | 1 |
| Gram Positive | Staphylococcus aureus | 4 |
|  | Enterococcus | 6 |
|  | Streptococcus agalactiae | 3 |
|  | Streptococcus dysgalactiae | 4 |
|  | Streptococcus pneumoniae | 2 |
|  | Streptococcus pyogenes | 2 |

**Table S3:**

| Number of patients with each viral species | | |
| --- | --- | --- |
| Enterovirus | Enterovirus PCR - CSF | 9 |
|  | Enterovirus RT-PCR | 1 |
| Herpes simplex | Herpes simplex Ag immunofluorescence | 1 |
|  | Herpes simplex type 1 PCR - CSF | 1 |
|  | HSV-1 PCR - CSF | 2 |
| Varicella zoster | Herpes zoster Ag immunofluorescence | 1 |
|  | Varicella zoster PCR | 2 |
|  | Varicella zoster virus identification with biopsy | 2 |
| Influenza A | Influenza A PCR positive | 1 |
|  | Influenza A RT-PCR | 1 |
|  | Influenza A RT-PCR - nasal fluid | 19 |
| Influenza B | Influenza B PCR - CSF | 1 |
|  | Influenza B RT-PCR | 2 |
|  | Influenza B RT-PCR - nasal fluid | 2 |
| Influenza | Influenza virus RT-PCR | 7 |
| Measles | Measles Ab IgM-B | 1 |
| Parainfluenza | Parainfluenza type 3 PCR | 2 |
| RSV | RSV (respiratory syncytial virus) RT-PCR | 6 |
| West Nile | West Nile virus IgM - CSF | 1 |

**Table S4:**

|  | **Age groups** | | | | |
| --- | --- | --- | --- | --- | --- |
|  | **Age <= 79 (years)** | | **Age 80+ (years)** | | **MW p value** |
|  | **N** | **CRPv (mean ± SD)** | **N** | **CRPv (mean ± SD)** |  |
| **Viral** | 42 | 0.89±1.3 | 20 | 0.96±1.04 | 0.567 |
| **Bacterial** | 28 | 4.23±2.97 | 46 | 4.52±2.6 | 0.696 |
| **Total** | 70 | 2.23±2.68 | 66 | 3.45±2.76 | 0.003 |
| N -total number of patients, MW p value of the Mann–Whitney test, CRPv- the difference between the first and second CRP tests divided by the time difference between them | | | | | |

**Table S5:**

|  | **Genders** | | | | |
| --- | --- | --- | --- | --- | --- |
|  | **Males** | | **Females** | | **MW p value** |
|  | **N** | **CRPv (mean ± SD)** | **N** | **CRPv (mean ± SD)** |  |
| **Age <= 79 (years)** | 41 | 2.19±2.79 | 29 | 2.9±2.56 | 0.844 |
| **Age 80+ (years)** | 27 | 3.24±3.00 | 39 | 3.59±2.62 | 0.445 |
| **Total** | 68 | 2.6+2.9 | 68 | 3.04±2.66 | 0.238 |
| N -total number of patients, MW p value of the Mann–Whitney test, CRPv- the difference between the first and second CRP tests divided by the time difference between them | | | | | |

**Table S6:**

|  | **Genders** | | | | | |  |
| --- | --- | --- | --- | --- | --- | --- | --- |
|  | **Males** | | **Females** | | **MW p value** |  |  |
|  | **N** | **CRPv (mean ± SD)** | **N** | **CRPv (mean ± SD)** |  |  |  |
| **Viral** | 41 | 2.19±2.79 | 29 | 2.9±2.56 | 0.844 |  |  |
| **Bacterial** | 27 | 3.24±3.00 | 39 | 3.59±2.62 | 0.445 |  |  |
| **Total** | 68 | 2.6+2.9 | 68 | 3.04±2.66 | 0.238 |  |  |
| N -total number of patients, MW p value of the Mann–Whitney test, CRPv- the difference between the first and second CRP tests divided by the time difference between them. | | | | | | | |
